# The replication protein of duck circovirus unwinds dsDNA in an ATP-driven and metal ion-dependent manner from 3′ to 5′

**DOI:** 10.3389/fvets.2025.1679348

**Published:** 2025-09-30

**Authors:** Lihan Tao, Chengcheng Wu, Na Li, Cui Lin, Jia Tan, Jianzhen Huang

**Affiliations:** ^1^College of Animal Science and Technology, Jiangxi Agricultural University, Nanchang, China; ^2^Institute of Animal Husbandry and Veterinary Medicine, Jiangxi Academy of Agricultural Sciences, Nanchang, China

**Keywords:** duck circovirus, replication protein, ATPase, unwinding, antiviral drugs

## Abstract

Duck circovirus (DuCV) causes immunosuppression, co-infection and increased mortality in ducks, and the prevalence and infection rate of DuCV have risen in recent years, resulting in significant economic losses to the duck industry. Studying the mechanism of virus replication is particularly important for controlling DuCV infection. The replication protein (Rep) encoded by the DuCV genome is a highly conserved enzyme and plays key roles in viral replication, making it an ideal target for antiviral drugs. However, the biochemical characteristics of DuCV Rep were still unknown. Here, we utilized the prokaryotic expression system to express the Rep, and the protein was purified and identified. Then, Rep’s activities and their influencing factors were explored by a series of experiments *in vitro*. The results showed that Rep had the enzyme concentration-dependent ATPase and unwinding activities, and dual functions of Rep were both affected by the type and concentration of divalent metal ions. Moreover, Rep was able to use the energy generated from the hydrolysis of any nucleoside triphosphates (NTPs) to unwind double-stranded DNA (dsDNA). Analogs and hydrolysates were unable to replace ATP for Rep to exert the unwinding activity. We also found that the 3′-terminal single-strand extension (3′-overhang) of the substrate was indispensable for the unwinding activity of Rep. The results indicated that Rep had a 3′–5′ unwinding directionality, and Rep was capable of unwinding dsDNA with a 3′-overhang of not less than 1 nt. In addition, the efficiency of the Rep-catalyzed unwinding reaction increased with the increase of the length of the 3′-overhang, but slightly decreased with the increase of the length of the duplex region. In this study, we systematically characterized the dual activities of DuCV Rep, which was conducive to deeply understanding the molecular mechanism of Rep in regulating virus replication and the development of antiviral drugs.

## 1 Introduction

Duck circovirus (DuCV) is a single-stranded circular DNA virus without an envelope that belongs to the genus *Circovirus* of the family *Circoviridae* (1). DuCV is widespread in domestic duck populations and almost all breeds of ducks are susceptible (2). The transmission of DuCV primarily occurs through horizontal and potential vertical routes (3). DuCV infection mainly causes lymphopenia, atrophy, and histiocyte hyperplasia in ducks. Ducks are immunosuppressed after infection, and the typical clinical symptoms are messy feathers, growth retardation and weight loss. And DuCV is often mixed with other viruses, which brings great losses to the duck industry (4). There is currently a lack of effective vaccines or prevention strategies for DuCV infection, which makes it difficult to control the spread of this virus in the waterfowl farming industry. Moreover, as there is currently no suitable *in vitro* culture system for DuCV, it is difficult to conduct etiological research and virus isolation of DuCV (5).

The genome of DuCV is approximately 2 kb in length and comprises three open reading frames (*ORFs*), including *ORF1*, *ORF2*, and *ORF3* (6). *ORF1* encodes a replication protein (Rep), and *ORF2* encodes the major immunogenic capsid protein. The Rep of DuCV plays an important role in the replication of DuCV, and the amino acid sequences of Rep are highly conserved in various genotypes (5). Therefore, Rep is also considered an ideal target for the development of anti-circovirus drugs. Moreover, the nuclear localization signal (NLS) of Rep at the N-terminal residue 10–37 has a nuclear localization function (7). After entering the cell nucleus, the single-stranded circular DNA of the virus first transcribes the Rep protein, and then the double-stranded replicates are synthesized by the cooperation of multiple factors such as Rep and DNA polymerase in the host cell.

The Rep protein of circovirus is a critical and multifunctional replication protein responsible for initiating replication of the viral genome, and this process is usually the rolling circle replication (8). Rep has a DNA-binding activity to recognize and bind replication origins on the viral genome. Rep also possesses a nuclease activity that cleaves the viral DNA strand at the specific sites to form a free 3′-OH terminus, which serves as a primer for DNA synthesis, and the process of DNA cleaving is a significant step in the initiation of rolling circle replication (9). In addition, the Rep of some circoviruses also has the ligase activity to link the newly synthesized linear genome during the later stages of replication (8).

Helicase is a protein that is widely present in almost all living forms in nature. Most viruses use host helicases for replication, and some viruses can directly encode helicases. Helicase activity (also named the unwinding activity) is one of the core functions of Rep of circoviruses, and Rep can utilize the energy provided by ATP hydrolysis to unwind the double-stranded DNA (dsDNA) in front of the replication fork and provide a single-stranded template for DNA synthesis (9). The nucleoside triphosphatase activity of Rep, also known as ATPase activity, hydrolyzes ATP to provide energy for helicase activity and other functions (10, 11). However, the regulatory molecular mechanism of Rep protein of duck circovirus remains unclear, and the biochemical characterization of Rep activities is lacking.

In this study, the high-purity recombinant Rep protein of DuCV was successfully obtained through the prokaryotic expression system, and the ATPase and unwinding activities of Rep were explored through multiple experiments *in vitro*. Furthermore, the factors that regulate Rep activities were also analyzed. These findings will deepen the understanding of the replication mechanism of circovirus, and provide a theoretical basis for the development of antiviral strategies targeting Rep protein.

## 2 Materials and methods

### 2.1 Recombinant plasmid construction and protein expression

The sequences of *Rep* gene of DuCV isolate Jiangxi/GZ-GXG0919/2022 (GenBank accession number: OR387756.1) were downloaded from the National Center for Biotechnology Information (NCBI) database, and the *Rep* gene was synthesized by Tsingke Biotechnology (Beijing, China). The Rep gene was inserted into the empty vector pSmart1 by homologous recombination, and the amino-terminal of the vector contained a His-SUMO tag, which was used for the fusion expression and purification of the target protein. Then, the recombinant plasmid pSmart1-Rep was transformed into BL21 (DE3) competent cells, and the bacterial liquid was spread onto LB plates and incubated at 37°C overnight. Monoclonal colonies were picked up and placed in LB liquid medium containing kanamycin. We used the control variable method to explore the optimal conditions for protein expression, including temperature, inducer concentration and shaker speed. Finally, 3 L of the bacterial liquid was expanded and cultured at 37 C, and 0.2 mM IPTG was added when the OD_600_ value of the bacterial liquid reached 0.6 ~ 0.8. The bacterial liquid was cultured at 220 rpm in a shaker at 15°C for 16 h, and the bacterial liquid was centrifuged at 5,000 r/min for 15 min, and the bacterial sediment was collected.

### 2.2 Purification and identification of DuCV rep

The harvested bacterial sediment was resuspended in ice-cold Buffer A (500 mM NaCl, 25 mM Tris–HCl and 1 mM PMSF, pH = 8.0), and the ultrasonic disruption was performed by using an ultrasonic crusher (Scientz, Ningbo, China). The mixture solution was centrifuged at 14,000 r/min for 30 min, and the supernatant was collected and filtered through a 0.45 μm disposable syringe filter (Millipore, United States). The supernatant was incubated with Ni^2+^-NTA-agarose at 4°C overnight, and eluted with a linear gradient concentration of imidazole from 20 to 500 mM. The purified products were centrifuged in an ultrafiltration centrifugal tube (Millipore, United States), and the buffer was replaced with Buffer B (200 mM NaCl, 200 mM Tris–HCl and 10% glycerol). The protein purity was evaluated using Coomassie brilliant blue 250 staining, and the protein identity was analyzed by Western blotting. The concentration of purified protein was determined using a Detergent Compatible Bradford Protein Assay Kit (Beyotime, Beijing, China). Purified protein was divided into 20 μL/vial and stored at −80 C. All steps of protein purification were performed at 4 C.

### 2.3 Preparation of duplex substrates

The 5′-carboxyfluorescein (5′-FAM)-labeled and unlabeled single-stranded DNA (ssDNA) were synthesized by Tsingke Biotechnology (Beijing, China) and purified by HPLC. The DNA sequences used in this study were all referred to the genome of duck circovirus shown in Table 1. The dsDNA for the unwinding experiment was prepared by the annealing reaction of two ssDNA *in vitro*. To generate the dsDNA, two complementary ssDNA of the same molar concentration were added to the annealing buffer (50 mM Tris and 50 mM NaCl), and then the mixture was heated in a metal bath at 95 C for 5 min, and the metal bath was placed in a sealed foam box and transferred to 4 C overnight. The annealing products were diluted to a specific concentration and detected by the native PAGE, and the gels were placed into the multifunctional laser scanner (Cytiva, United States) for imaging. The absence of clearly visible ssDNA in the lanes indicated that the annealing reaction was complete, and the products were stored at −20°C.

**Table 1:** Oligonucleotide sequences used in this study.

Name	Sequence (5′–3′)	nt
D1	TGGGCAGCTGGTTACAGCTG	20
D2	*FAM-*CAGCTGTAACCAGCTGCCCA	20
D3	GATATACACTTGGGCAGCTGGTTACAGCTG	30
D4	TGGGCAGCTGGTTACAGCTGGTTATGGTGA	30
D5	TGGGCAGCTGGTTACAGCTGG	21
D6	TGGGCAGCTGGTTACAGCTGGT	22
D7	TGGGCAGCTGGTTACAGCTGGTT	23
D8	TGGGCAGCTGGTTACAGCTGGTTA	24
D9	TGGGCAGCTGGTTACAGCTGGTTAT	25
D10	TGGGCAGCTGGTTACAGCTGGTTATGGTGA	30
D11	TGGGCAGCTGGTTACAGCTGTGTCACGACAGTTATGGTGA	40
D12	*FAM-*TGTCGTGACACAGCTGTAACCAGCTGCCCA	30
TrapD2	CAGCTGTAACCAGCTGCCCA	20
TrapD12	TGTCGTGACACAGCTGTAACCAGCTGCCCA	30

### 2.4 Measurement of the ATPase activity of DuCV rep

We used the commercial kinase activity assay kits (Beyotime, Shanghai, China) to determine the ATPase activity of DuCV Rep, and the entire assay process consists of two reactions. Firstly, partial ATP in the reaction buffer was hydrolyzed by Rep into ADP and inorganic phosphoric acid. A 5 μM ATP was added to the reaction buffer (30 mM Tris–HCl, 3 mM MgCl_2_ and 2 mM DTT) and transferred to the 96-well plate, and the reaction of ATP hydrolysis was initiated by adding Rep and incubated at room temperature for a specified time. Then, the remaining ATP was involved in the luciferin oxidation reaction catalyzed by luciferase. After consuming ATP and oxygen, oxidized luciferin was generated and fluorescence was released. We were able to determine the strength of ATPase activity of Rep based on the magnitude of the luminescence value. The larger the luminescence value, the more ATP remaining in the Rep-catalyzed ATPase reaction, and the weaker the ATPase activity of Rep. In this study, to clarify the effect of divalent metal ions on Rep’s ATPase activity, the EDTA controls were introduced to chelate metal ions in the reaction system. Specifically, prior to the reaction, 10 mM EDTA·Na_2_ was added to the reaction buffer containing 3 mM metal ions (MgCl_2_, MnCl_2_, CaCl_2_, or ZnCl_2_). After incubation at room temperature for 10 min, the Rep was then added to initiate the reaction. When the reaction time was reached, an equal detection reagent was added to the reaction mixture and incubated at room temperature for 10 min, and the fluorescence released during the reaction was detected by a multifunctional microplate reader (Molecular Devices, San Jose, CA, United States). The bar charts shown in this paper were plotted by the Origin 9.0 software. Data were derived from three independently replicated experiments and were expressed as mean ± standard deviation.

### 2.5 PAGE-based unwinding experiments for DuCV rep

In this study, the unwinding experiments of duck circovirus Rep were carried out by two strategies, endpoint detection and time titration. The former refers to the detection of the unwinding products when the reaction time reaches 30 min, and the latter refers to the determination of the unwinding products at different time points in the range of 0–20 min. The substrates used in the study included dsDNA with various terminal structures and different lengths of double-stranded regions. Before the unwinding reaction, 16 nM dsDNA and 3 mM ATP were mixed in the unwinding buffer (30 mM Tris–HCl, 0.1 mg/mL BSA, 1% glycerol, 1 μM trap DNA, 3 mM MgCl_2,_ and 2 mM DTT) and incubated at 25°C for 5 min, and then 250 nM Rep was added as the start of the reaction. Here, the trap DNA was used to competitively bind to the non-fluorescently labeled ssDNA to prevent the annealing of ssDNA in the unwinding products, and the sequences of trap DNA were the same as the fluorescently labeled ssDNA. Additionally, we established experimental groups incorporating EDTA·Na_2_. Here, 10 mM EDTA·Na_2_ was added to the reaction buffer containing 3 mM metal ions (MgCl_2_, MnCl_2_, CaCl_2,_ or ZnCl_2_) and incubated at 25°C for 10 min, and then Rep was introduced to initiate the unwinding reaction. The dsDNA was opened to ssDNA as a positive control after denaturation at 95°C, and the reaction without Rep was used as a negative control. When the specific reaction time was reached, 10 μL reaction products were removed and added to the equal precooled stop buffer (50 mM EDTA·Na_2_, 1% SDS and 10% glycerol). The mixture was resolved on the native PAGE gel by electrophoresis at 150 V for 3 h, and then the bands were visualized using the Typhoon RGB multifunctional laser scanner (Cytiva, United States). ImageJ 1.48v was used to analyze the gray value of the bands and calculate the proportion of ssDNA in the unwinding product.

## 3 Results

### 3.1 Expression and purification of DuCV rep

**Figure 1:**
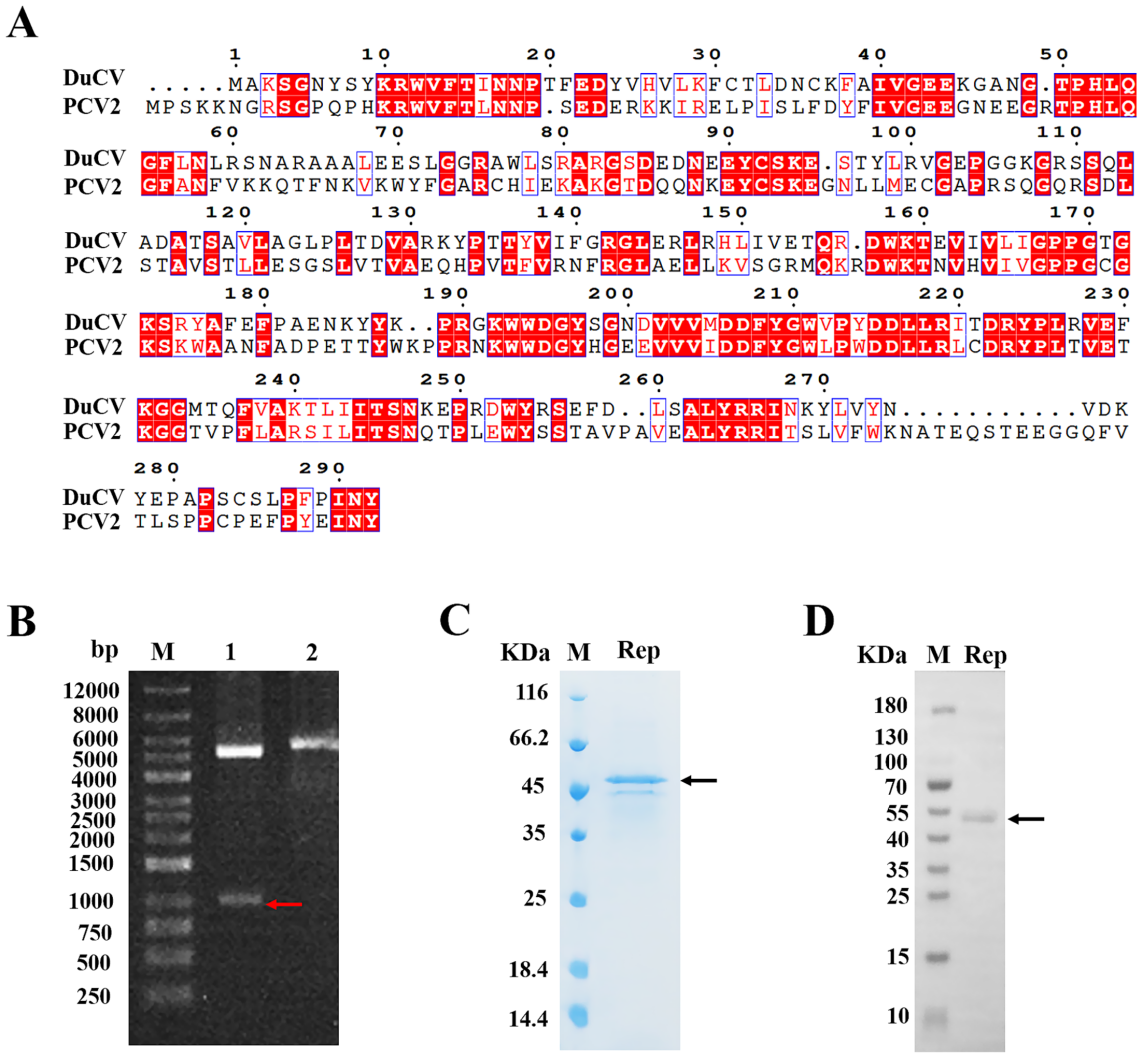
Expression and purification of the Rep protein of DuCV. **(A)** Alignment of the amino acid sequences of DuCV Rep and PCV-2 Rep. The sequences of Rep were derived from the NCBI database, including DuCV isolate Jiangxi/GZ-GXG0919/2022 (GenBank accession number OR387756.1) and PCV2 strain AY4844 (GenBank accession number KX814348.1). The Rep sequences of two circoviruses were blasted using the online programs CLUSTALW (https://www.genome.jp/tools-bin/clustalw) and ESPript 3.0 (https://espript.ibcp.fr/ESPript/cgi-bin/ESPript.cgi). **(B)** Identification of the recombinant plasmid pSmart1-Rep by enzyme digestion. Lane 1 indicated the electrophoresis of the digested recombinant plasmid, and lane 2 represented the electrophoresis of the undigested recombinant plasmid. **(C)** The purified Rep was analyzed by SDS-PAGE. **(D)** The purified Rep was identified by Western blotting. The letter “M” indicated the protein molecular weight marker, the red arrow denoted the band of gene Rep, and the black arrow represented the bands of the purified Rep.

Due to the lack of an effective cell culture system for DuCV, research on its mechanisms of infection, genome replication and virus maturation remained unknown. Currently, the studies of animal circoviruses were mainly of porcine circovirus (PCV), and PCV had three subtypes. However, the high pathogenicity of porcine circovirus type two (PCV2) resulted in significant economic losses of the pig industry, and this subtype had been studied the most deeply at present. Therefore, we compared the amino acid sequences of the Rep from DuCV and PCV-2, and the alignment results showed that the sequence identity of Rep was 41%, and the sequence similarity was 77% (Figure 1A). The results indicated that there was a significant difference in the Rep sequences of DuCV and PCV-2, and it was necessary to conduct research on DuCV Rep.

Here, the prokaryotic expression system of *Escherichia coli* was used to express the Rep protein of DuCV, and we firstly inserted the Rep gene into the vector pSmart1, and then identified the recombinant plasmid by enzyme digestion and DNA sequencing. As shown in Figure 1B, the bands in lane 1 after double enzyme digestion of the recombinant plasmid were subjected to agarose gel electrophoresis, and the positions of two bands were corresponded to the sizes of pSmart1 (5,594 bp) and Rep gene (879 bp), suggesting that the recombinant plasmid pSmart1-Rep was successfully constructed. To facilitate soluble expression and purification, the recombinant Rep protein was tagged with His and SUMO at the amino-terminus, and the molecular weight of the fusion protein was approximately 46 kDa. The target protein was eluted and collected by affinity chromatography, and relatively pure Rep protein was obtained (Figure 1C). Moreover, we also used Western blotting to identify the purified Rep, and the position of the protein band was consistent with the theoretical molecular weight of the target protein (Figure 1D).

### 3.2 DuCV rep protein exhibited a strong ability to hydrolyze ATP

**Figure 2:**
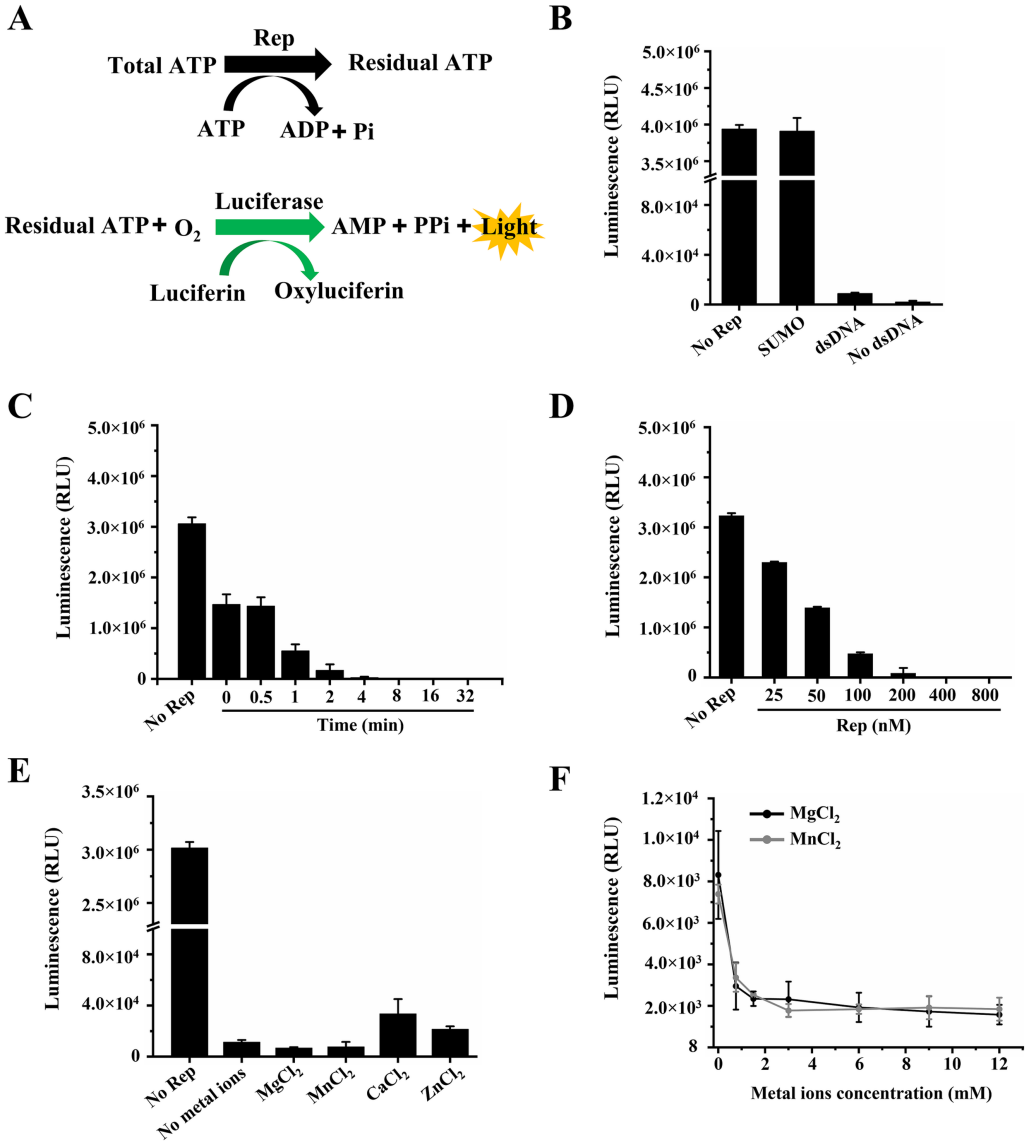
Determination of the ATPase activity of Rep. **(A)** Schematic diagram of the ATPase activity assay. **(B)** The dependence of the ATPase activity of Rep on DNA was tested. **(C)** Time titration experiment of ATP hydrolysis catalyzed by Rep. And the time points were 0, 0.5, 1, 2, 4, 8, 16, and 32 min. **(D)** Measurement of the ATPase activity under different concentrations of Rep. And the Rep concentrations were 0, 25, 50, 100, 200, 400, and 800 nM. **(E)** The impact of various metal ions on the ATPase activity of Rep was determined. **(F)** The effect of MgCl_2_ concentration on the ATPase activity of Rep was determined. And the MgCl_2_ concentrations were 0, 0.75, 1.5, 3, 6, 9, and 12 mM.

In general, helicases are known to require the energy from the hydrolysis of ATP to unwind nucleic acid substrates. The Rep protein of circoviruses also had the ability to hydrolyze ATP and separate dsDNA, and we measured the ATPase activity of DuCV Rep by the commercial kinase activity assay kits. The principles of ATPase assays were based on two steps: one was the ATP hydrolysis reaction catalyzed by Rep, and the other was the oxidation reaction of luciferin (Figure 2A). We determined Rep’s ATPase activity by measuring the remaining amount of ATP in the Rep-catalyzed reaction. The higher the ATPase activity of Rep, the less ATP remained, and it meant that less ATP was available for luciferin oxidation, resulting in lower luminescence detected by the instrument.

We first checked whether the ATPase activity of Rep depended on nucleic acid substrates, and the results showed that Rep has strong ATPase activity regardless of the presence of dsDNA in the reaction system (Figure 2B), indicating that Rep possessed DNA-independent ATPase activity. Moreover, to eliminate the influence of the SUMO protein on the experimental results, we confirmed that SUMO was unable to hydrolyze ATP, and the SUMO used here was from our laboratory preservation. We also observed the ATP hydrolysis catalyzed by Rep at different time points through time titration experiments, and the results showed that the luminescence decreased rapidly over time and reached the lowest value at 8 min, indicating that Rep had almost completely hydrolyzed the ATP in the system at this time (Figure 2C).

In addition, the effect of Rep concentration on ATPase activity was also determined, and various concentrations of Rep were added to the reaction buffer. We found that the luminescence was decreased with increasing the Rep concentrations, and reached the minimum level when the Rep concentration was 400 nM (Figure 2D). As we know, divalent metal ions are cofactors for helicases, and we compared the ATPase activities of Rep in the presence of MgCl_2_, MnCl_2_, CaCl_2,_ and ZnCl_2_. The results showed that Rep still exhibited strong ATPase activity in the absence of metal ions. The addition of MgCl_2_ or MnCl_2_ slightly enhanced this activity, whereas ZnCl_2_ or CaCl_2_ inhibited it (Figure 2E). In comparison, when EDTA·Na_2_ was incorporated into the reaction buffer, the chemiluminescence signals became nearly equivalent to those without metal ions, and the previously observed modulatory effects of metal ions on the Rep’s ATPase activity were almost disappeared (Supplementary Figure S1). Furthermore, the effect of metal ion concentrations on the ATPase activity of Rep was also examined in this study, and we found that Rep had strong ATPase activity at low concentrations (0–1.5 mM) of MgCl_2_ and MnCl_2_. Specifically, ATP was nearly completely hydrolyzed when the concentrations of either MgCl_2_ or MnCl_2_ reached 3 mM (Figure 2F).

### 3.3 DuCV rep unwound the duplex substrates in a 3′–5′ direction

**Figure 3:**
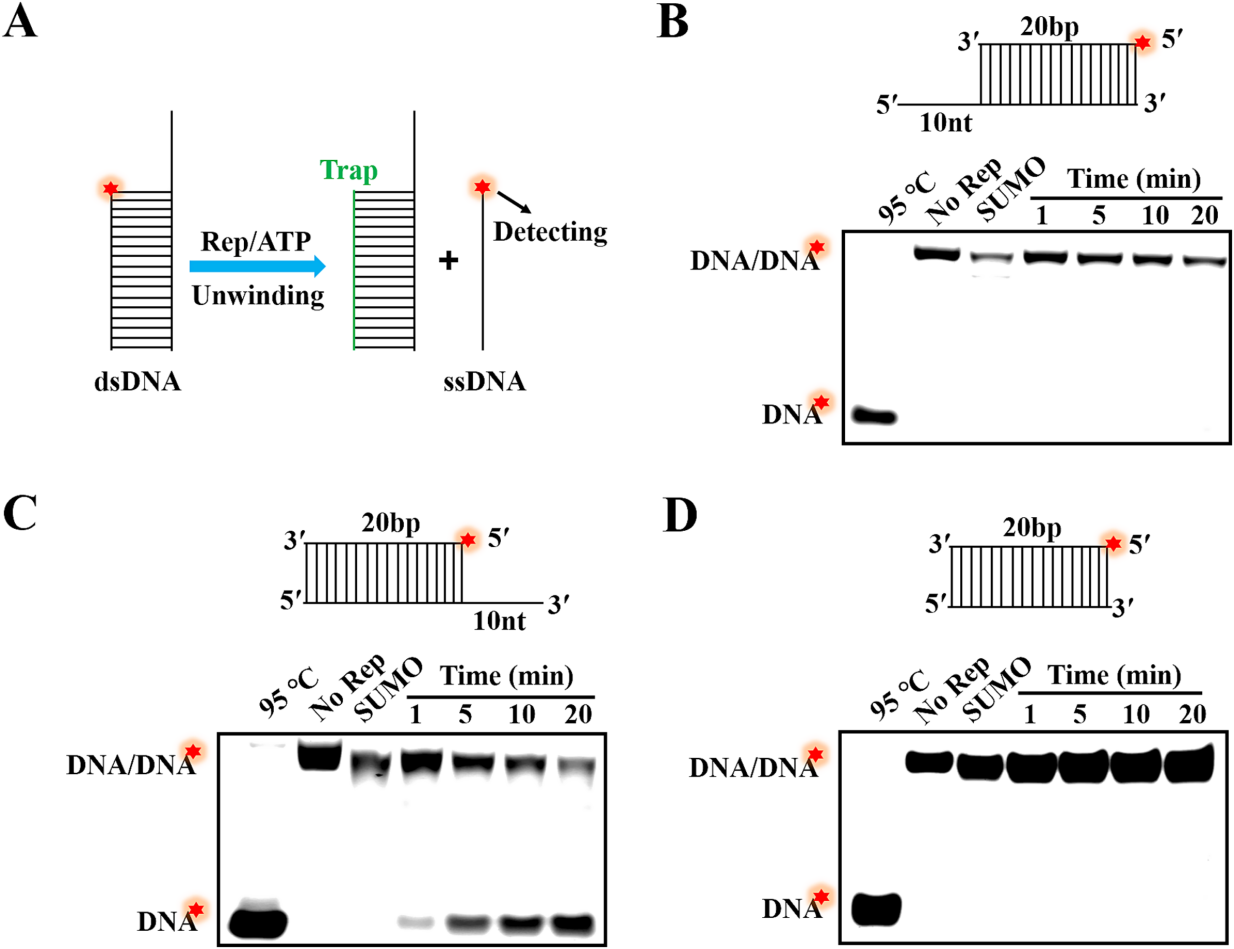
Determination of the unwinding directionality of DuCV Rep. **(A)** Schematic diagram of dsDNA unwinding by Rep. **(B)** Rep was unable to unwind the dsDNA with 5′-overhang. **(C)** The dsDNA with 3′-overhang was unwound by Rep. **(D)** Rep was incapable of unwinding the dsDNA without single-stranded extension. The Red stars in figures represented the 5′-FAM.

In the previous research, helicases mainly possessed two modes for separating of substrates: directional translocation unwinding and local unwinding. However, it was currently unknown whether the Rep protein of DuCV had directionality when unwinding substrates. In this paper, three different structures of dsDNA containing 5′-overhang, 3′-overhang and blunt end were formed by annealing of complementary ssDNA. The unwinding direction of Rep was identified by determination of dsDNA separation, and the schematic diagram of dsDNA unwinding was shown in Figure 3A. Here, dsDNA was denatured at 95°C to open into ssDNA as the positive controls, and the unwinding reactions without Rep were used as the negative controls. The results showed that the SUMO protein was incapable of unwinding any dsDNA, suggesting that the unwinding results we obtained were specifically derived from the Rep protein. We found that Rep was unable to unwind the dsDNA containing a 5′-overhang or blunt end (Figures 3B–D), but Rep was capable of unwinding the dsDNA with a 3′ -overhang, and the reaction products gradually increased over time (Figure 3C). The results indicated that the Rep of DuCV had a 3′–5′ direction in the unwinding activity.

### 3.4 Determination of the factors that regulate the unwinding activity of DuCV rep

**Figure 4:**
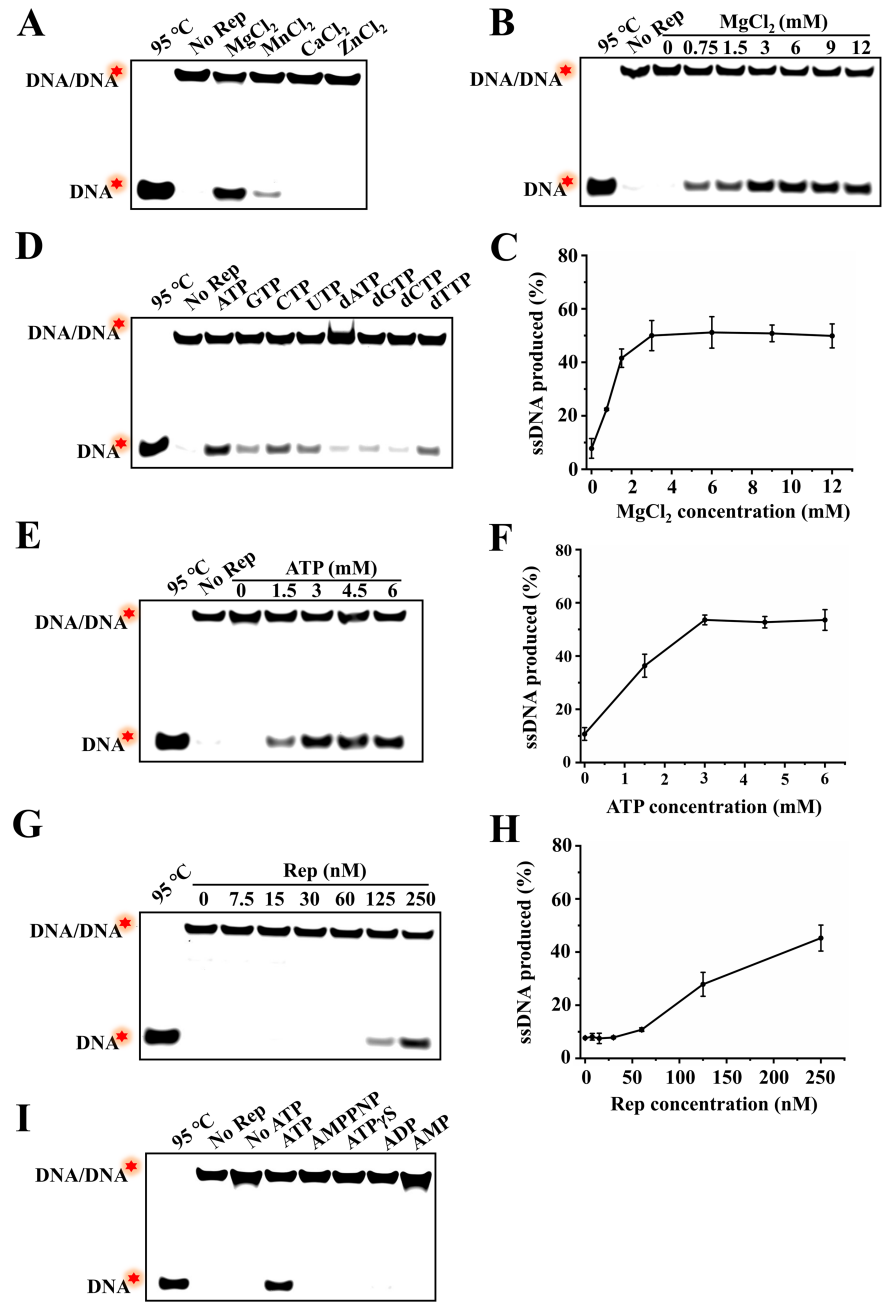
Identification of the factors regulating the unwinding activity of Rep. **(A)** Electrophoresis of unwinding products in the presence of different MgCl_2_, MnCl_2_, CaCl_2,_ and ZnCl_2_. **(B)** The effect of MgCl_2_ concentrations on the unwinding activity of Rep was detected, and the MgCl_2_ concentrations were 0, 0.75, 1.5, 3, 6, 9, and 12 mM. **(C)** Line graph of ssDNA yield as a function of MgCl₂ concentration. **(D)** Electrophoresis of unwinding products in the presence of NTPs and dNTPs. **(E)** The effect of ATP concentrations on the unwinding reaction was tested. And the ATP concentrations used in this assay were 0, 1.5, 3, 4.5, and 6 mM. **(F)** Line graph of ssDNA yield as a function of ATP concentration. **(G)** Determination of the effect of Rep concentrations on the unwinding activity, and the Rep concentrations were 0, 7.5, 15, 30, 60, 125, and 250 nM. **(H)** Line graph of ssDNA yield as a function of Rep concentration. **(I)** Electrophoresis of unwinding products in the presence of ATP and different substitutes. The red stars in the figures denoted the 5′-FAM.

To better characterize the unwinding activity of DuCV Rep, this study further investigated various factors influencing Rep unwinding activity. First, MgCl_2_, MnCl_2_, CaCl_2,_ and ZnCl_2_ were added to the unwinding reaction, and the proportion of dsDNA unwound was measured. The results showed that approximately 47% of dsDNA was unwound into ssDNA in the presence of MgCl_2_, and weak unwinding of dsDNA was observed with the involvement of MnCl_2_, while almost no product ssDNA was detected in the presence of CaCl_2_ and ZnCl_2_ (Figure 4A). Notably, the inclusion of excessive EDTA·Na_2_ to chelate metal ions completely abolished the unwinding activity of Rep, confirming that the observed activity was strictly dependent on the MgCl_2_ or MnCl_2_ (Supplementary Figure S2). Subsequently, the effect of MgCl_2_ concentrations on the unwinding activity of Rep was explored, and different concentrations of MgCl_2_ were added to the reaction buffer, and the ratio of product ssDNA generated was measured. The results showed that dsDNA was not unwound in the absence of MgCl_2_, and the unwinding efficiency of dsDNA was increased with the increase of MgCl_2_ concentrations, and reached a maximum of approximately 51% at 3 mM MgCl_2_ (Figures 4B,C). These results indicated that metal ion was essential for Rep to unwind dsDNA.

In addition, four types of NTPs (ATP, GTP, CTP, and UTP) and dNTPs (dATP, dGTP, dCTP, and dTTP) were added to the reaction buffer, and the corresponding unwinding products were measured. The results showed that Rep was able to utilize almost all NTPs and dNTPs to unwind dsDNA, but the unwinding activities were different, and Rep had the strongest unwinding activity in the presence of ATP (Figure 4D). We also investigated the effect of ATP concentration on the unwinding reaction catalyzed by Rep, and the results showed that without ATP, Rep was unable to unwind dsDNA without ATP, suggesting that the unwinding activity of Rep depended on the presence of ATP. Furthermore, the effects of ATP and Mg^2+^ concentrations on the unwinding reaction were similar, and the unwinding products gradually increased with the increase of ATP concentrations, reaching the maximum at 3 mM ATP. These results denoted that Rep required ATP hydrolysis to provide energy for the separating of dsDNA, and the Rep-catalyzed unwinding reaction exhibited ATP concentration dependence (Figures 4E,F). The unwinding reaction catalyzed by different concentrations of Rep was also measured, and it was found that the unwinding products were almost not observed when Rep concentration was in the range of 0–60 nM. And the unwinding efficiency was enhanced with the increase of Rep concentrations from 125 to 250 nM (Figures 4G,H).

Most helicases require ATP hydrolysis to provide energy for binding and unwinding dsDNA, and a few helicases only require ATP binding for the unwinding activity but not ATP hydrolysis, and some non-hydrolysable ATP analogs could be used in unwinding reactions (12). However, it is still unclear whether DuCV Rep had the ability to utilize ATP analogs to unwind dsDNA. To answer this question, we added ATP analogs (AMPPNP and ATPγS) and hydrolysis products (ADP and AMP) to the unwinding reaction to investigate whether these substances could replace ATP for the unwinding of dsDNA. The results showed that the dsDNA was only unwound by Rep in the presence of ATP, and AMPPNP, ATPγS, ADP, and AMP were all not used in the unwinding reaction. These results implied that the unwinding activity of Rep was strictly dependent on the primary hydrolysis reaction of ATP (Figure 4I).

### 3.5 Rep can unwind dsDNA containing a 3′-overhang with a length of at least 1 nt

**Figure 5:**
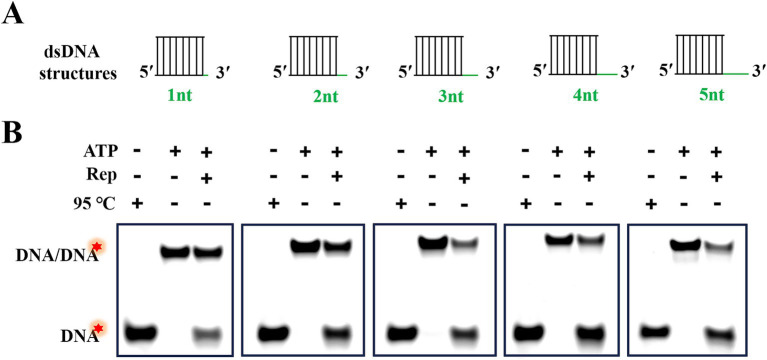
Rep-catalyzed unwinding reactions of dsDNA with various lengths of 3′-overhangs. **(A)** Schematic diagram of dsDNA structures with 3′-overhang lengths of 1, 2, 3, 4, and 5 nt. **(B)** Electrophoresis of reaction products of dsDNA with different 3′-overhangs. Green lines represented the 3′-overhangs, and red stars indicated the 5′-FAM labeled on the substrates.

According to Figure 3, we had demonstrated that Rep was able to unwind the dsDNA with a 3′-overhang, but the minimum length of the 3′-overhang needed for Rep to perform the unwinding activity was still unknown. To address this question, we prepared dsDNA containing five different lengths (from 1 to 5 nt) of 3′-overhangs via annealing reactions, and the structures of these dsDNA were shown in Figure 5A. We measured the products generated from the Rep-catalyzed unwinding reactions, and the dsDNA denatured at 95 C served as the positive control, and the unwinding reaction without Rep served as the negative control. The reaction was carried out at 37°C for 30 min, and the reaction products were separated on the native PAGE gels. We found that Rep was able to unwind all dsDNA with 3′-overhang lengths ranging from 1 to 5 nt into ssDNA (Figure 5B). The results indicated that the unwinding activity of Rep had no strict requirement for the 3′-overhang length of the dsDNA, and Rep was capable of unwinding dsDNA as long as it contained a 3′-overhang.

### 3.6 The lengths of 3′-overhangs and duplex regions of substrates exhibited opposite effects on the unwinding efficiency

**Figure 6:**
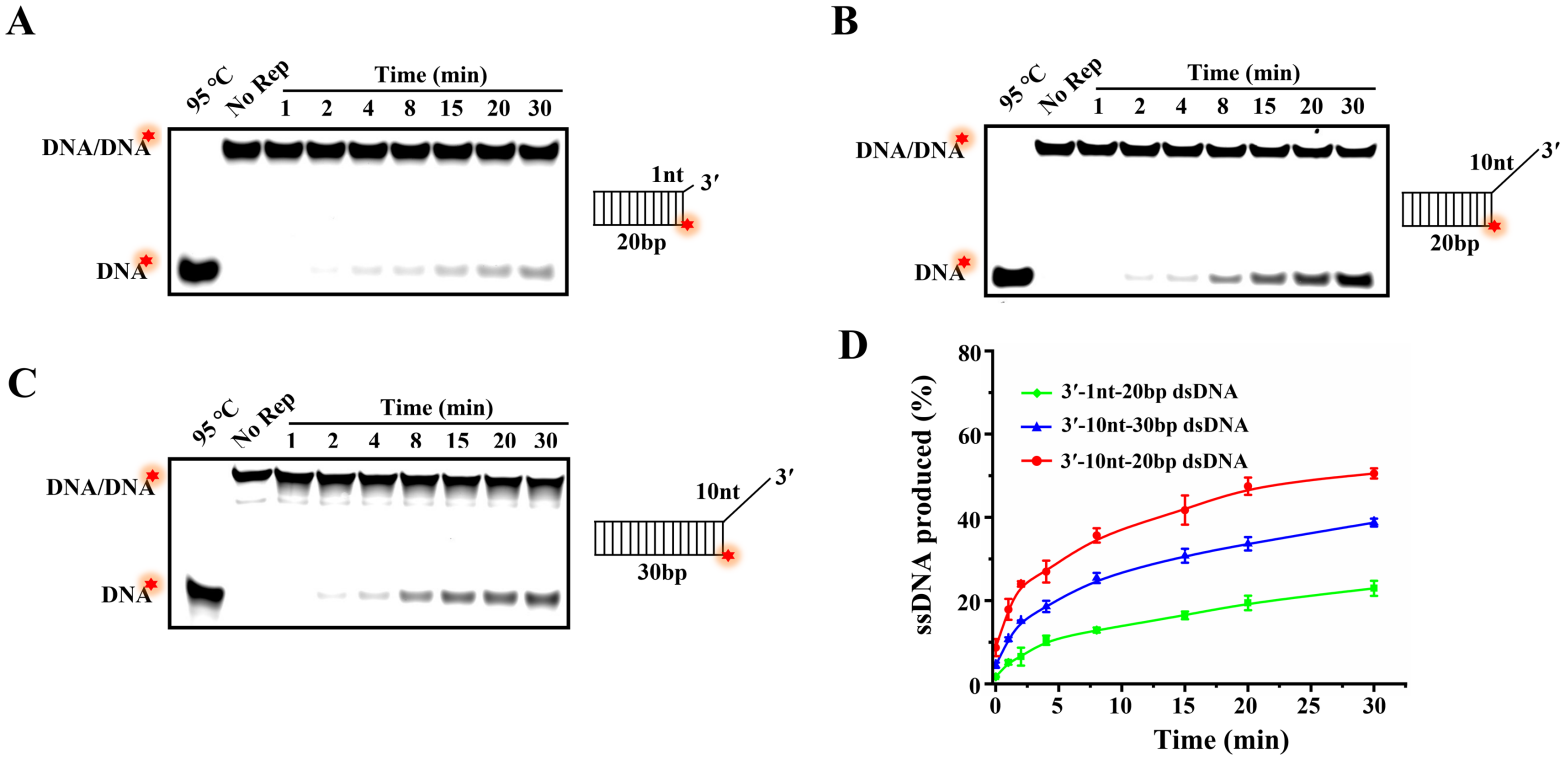
The effect of the lengths of 3′-overhang and duplex region on the unwinding efficiency. **(A)** Unwinding products of 20 bp dsDNA with a 1 nt 3′-overhang at different time points. **(B)** Unwinding products of 20 bp dsDNA with a 10 nt 3′-overhang at various time points. **(C)** Unwinding products of 30 bp dsDNA with a 10 nt 3′-overhang at different time points. **(D)** The curves of the proportion of products ssDNA vary with time. The red stars of the substrates represented the 5′-FAM.

In our previous study, we had revealed that Rep was able to use the energy from ATP hydrolysis to load and move along the 3′-overhang to unwind dsDNA in a 3′–5′ direction, but whether the 3′-overhang length affects the unwinding efficiency needed further study. Here, the unwinding reactions of dsDNA with 3′-overhangs of 1 and 10 nt were conducted, and the proportions of products ssDNA at different time points were fitted based on the results of time titration experiments (Figures 6A,B). The results showed that the ssDNA yields were all gradually increased over time, but the unwinding amplitude of dsDNA with a 1 nt 3′-overhang reached 23%, while the dsDNA with a 10 nt 3′-overhang was increased to 51% at the same time point (Figure 6D). The results elucidated that the efficiency of Rep-catalyzed unwinding reaction was increased with the increase of 3′-overhang lengths of dsDNA.

Different helicases exhibit varying abilities to move and unwind nucleic acid substrates, and the length of the duplex regions of substrates typically influences the helicase processivity. In order to explore the continuous unwinding activity of Rep, we compared the unwinding amplitudes of dsDNA with the duplex regions of 20 bp (Figure 6B) and 30 bp (Figure 6C). Compared to the unwound proportion of 20 bp dsDNA (51%), we found that 30 bp dsDNA was decreased to 39%, and the results indicated that the unwinding efficiency was decreased with the increase of the lengths of duplex regions of dsDNA (Figure 6D).

## 4 Discussion

Duck circovirus is a common pathogen that causes immune suppression in ducks and co-infects with other pathogens, resulting in significant economic losses to the poultry industry (13). The circovirus genome encodes the Rep protein with multiple functions, and the amino sequences of Rep of various circoviruses are conserved (14). In the previous research, the Rep protein of PCV1 possessed nucleic acid endonuclease and ATPase activities, and it was able to cleave and ligate viral ssDNA and played a crucial role in the initiation and termination of viral replication (15, 16). The Rep protein of PCV2 was confirmed to have the two activities of PCV1 Rep, but also have the helicase activity (17–19). Currently, the main strategies for preventing and controlling circovirus are the development of vaccines and drugs, and Rep is a potential target for antiviral drugs. In this study, we established the assay systems to detect the ATPase and unwinding activities of DuCV Rep *in vitro*, and the dual activities of Rep and the factors influencing these activities were analyzed. Notably, the SUMO-only control was also established, and we confirmed that SUMO lacked the ATPase and unwinding activities, indicating that all activities we observed were specifically derived from the Rep, the SUMO of the fusion protein Rep was unable to alter the conclusions of this study. However, the SUMO tag may affect the activities of Rep by influencing enzyme conformation or flexibility, and we need to use SUMO proteases for precise cleavage to remove SUMO tag in future studies.

**Figure 7:**
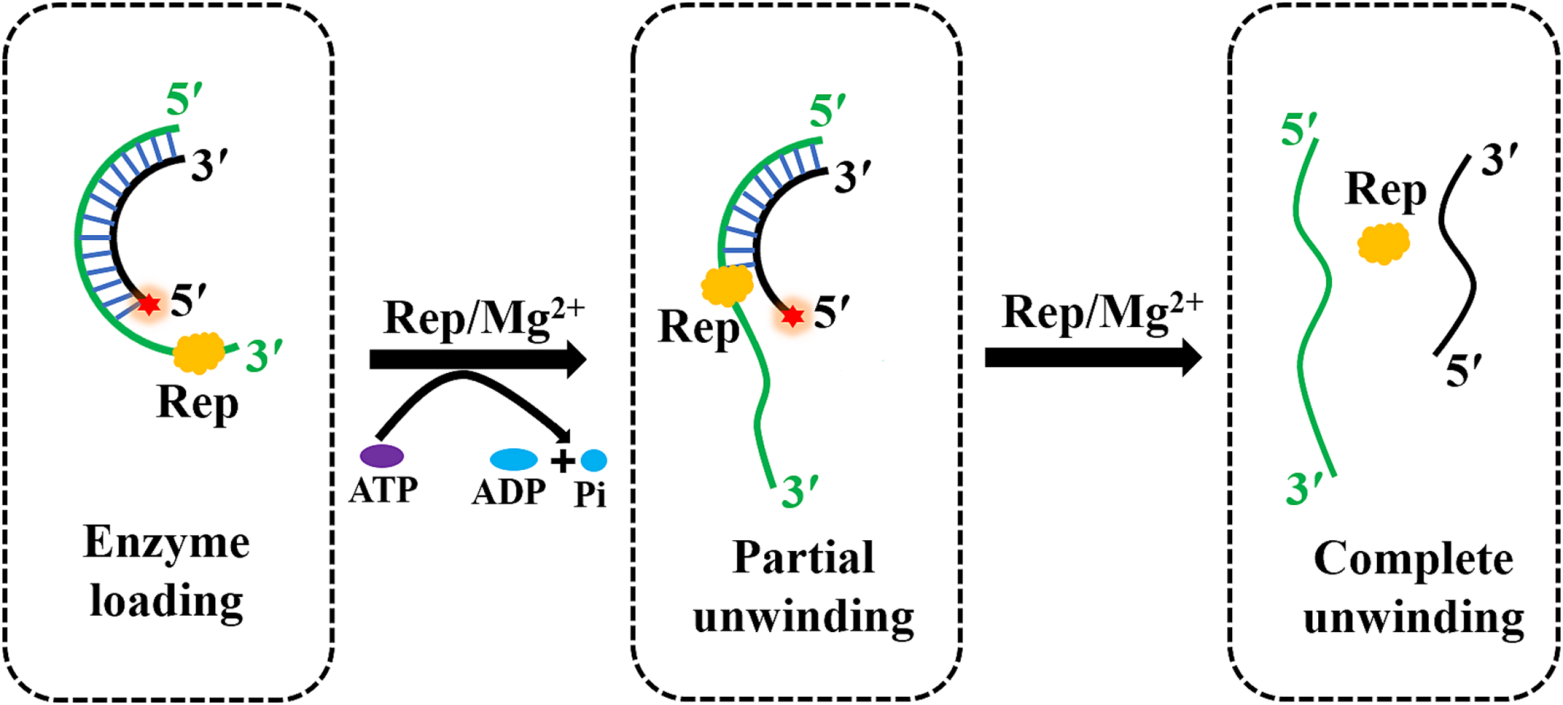
The model of the DuCV Rep-catalyzed unwinding reaction. Rep played an important role in the rolling circle replication of DuCV, and Rep had the ability to unwind duplex substrates by using the energy from ATP hydrolysis in a unidirectional and Mg^2+^-dependent manner. The green line indicated the loading strand with a 3′-overhang for Rep, and the black strand was the complementary strand of the loading strand. The red star of the substrate represented the 5′-FAM.

Helicases are the molecular motor proteins that regulate the gene replication, and helicases are able to utilize the energy released by ATP hydrolysis to separate the duplex nucleic acids and generate the replication templates (20–22). In previous studies, a few helicases opened double strands by the local unwinding manner and lacked the unwinding directionality, and most helicases first loaded onto the single-stranded extension and then moved in a specific direction to unwind the duplex substrates. The superfamily two vaccinia viral helicase nucleoside triphosphate phosphohydrolase-II (NPH-II) exhibited the unwinding activity with a 3′–5′ polarity in an ATP-dependent manner (23). However, DDX3X was shown as an ATP-independent triplex DNA helicase to unwind substrates with a 5′–3′ direction to prevent DNA damage (24). In our study, the Rep protein of DuCV was shown to hydrolyze ATP in a magnesium ion-dependent fashion, and Rep was capable of using the energy from ATP hydrolysis to unwind dsDNA in a 3′–5′ direction (Figure 7). And the Rep of PCV2 also exhibited an ATP-dependent and unidirectional unwinding activity, which was basically consistent with the DuCV Rep (10).

The catalytic functions of enzymes are often regulated by multiple factors, including metal ions, temperatures and the concentrations of substrates and enzymes, and so on. This study investigated the factors that influenced the ATPase and helicase activities of DuCV Rep protein, and the results revealed that Rep exhibited time- and enzyme concentration-dependent ATPase activity. Specifically, while metal ions were not essential for ATPase activity, their addition to the reaction solution can enhance Rep’s ATPase activity. In contrast, the Rep protein of beak and feather disease virus (BFDV), which also belonged to the circovirus family as DuCV, exhibited Mg^2+^-dependent ATPase activity (11). Compared to Rep’s ATPase activity, metal ions and ATP were essential for Rep to exert its unwinding activity. Within a concentration range below 3 mM, the unwinding activity of Rep exhibited dependence on Mg^2+^ and ATP concentrations. Additionally, it was found that non-hydrolyzable ATP analogs such as ATPγS and AMPPNP, as well as ATP hydrolysis products ADP and AMP, could not replace ATP in enabling Rep to exert its ability to unwind dsDNA. However, the DEAD-box family helicase Ded1p was able to utilize the non-hydrolysable ATP analog ADP-beryllium fluoride to unwind dsRNA, and ATP hydrolysis was required for fast enzyme release from the substrates and enzyme recycling (12). The helicase CMG of *Saccharomyces cerevisiae* consisted of Cdc45, MCM and GINS subunits, and CMG was capable of using ATPγS to fuel unwanted DNA unwinding during the helicase preloading step, but AMPPNP enabled CMG loading onto DNA without unwinding (25).

The 3′-overhang of substrates was essential for the unwinding activity of DuCV Rep, and we also determined the minimum length of the 3′-overhang required for Rep to unwind dsDNA. Here, dsDNA with different lengths of 3′-overhang (from 1 to 5 nt) was used in the unwinding experiments, and the duplex regions of dsDNA were all 20 bp. Interestingly, the results revealed that the dsDNA with a 3′-overhang length of at least 1 nt was able to be unwound by Rep, and the efficiency of the unwinding reaction catalyzed by Rep increased with the length of the 3′-overhang. In addition, we also tested the effect of the lengths of duplex regions on the unwinding efficiency, and the results demonstrated that the efficiency decreased slightly as the duplex region lengths increased. This phenomenon may be attributed to two factors: first, the limited processivity of Rep, which constrains its capacity for sustained unwinding of extended DNA regions; second, the enhanced structural stability of longer dsDNA due to a greater number of complementary base pairs, which imposes thermodynamic and kinetic barriers to Rep-driven duplex separation.

The Rep protein is a key protein that regulates the rolling circle replication of circoviruses, and it can recognize and cleave the stem-loop structure of the viral genome to initiate viral replication. In the previous studies, the key amino acid mutations in the Rep protein of PCV1 reduced the level of viral replication (26). Besides, C-terminal truncation mutations at the carboxyl terminus of the Rep protein of PCV2 reduced the viral replication and infectivity (27). However, this paper has not yet identified the key amino acid sites in Rep, which can be studied in future research. However, the key amino acid sites of DuCV Rep had not been identified in this study, and we decided to conduct further research in the subsequent experiments.

Virus-encoded helicases are indispensable in the viral life cycle. Screening drugs from the compound library that can bind to specific sites of helicases, thereby inhibit viral replication by suppressing ATP hydrolysis or unwinding activity, has become one of the most important strategies for the development of antiviral drugs (28, 29). The natural compound ZINC12899676 was able to directly target the ATPase activity region of the PEDV helicase Nsp13, and the conformation and function of Nsp13 were changed for inhibiting viral replication (30). Ranitidine bismuth citrate (RBC) inhibited the dual ATP hydrolysis and unwinding activities of SARS-CoV-2 Nsp13 in a dose-dependent manner. Moreover, RBC had relatively low cytotoxicity and inhibited SARS-CoV-2 replication, reduced viral load, and alleviated pneumonia symptoms (31). Guanidine hydrochloride was shown to inhibit the NTPase and unwinding activities of the VP35 protein of Ebola virus (EV), thereby reducing the viral replication and transcription (32). To date, there is currently a lack of screening for inhibitors of DuCV Rep, which is also a key focus of our future research.

## Data Availability

The raw data supporting the conclusions of this article will be made available by the authors, without undue reservation.
