# Supplementary material for: The replication protein of duck circovirus unwinds dsDNA in an ATP-driven and metal ion-dependent manner from 3′ to 5′
Source: Front Vet Sci. 2025 Sep 30;12:1679348. doi: 10.3389/fvets.2025.1679348 (PMC12518095; doi:10.3389/fvets.2025.1679348)
Supplement: Supplementary file 1 [file Image_1.pdf]

Supplementary Material

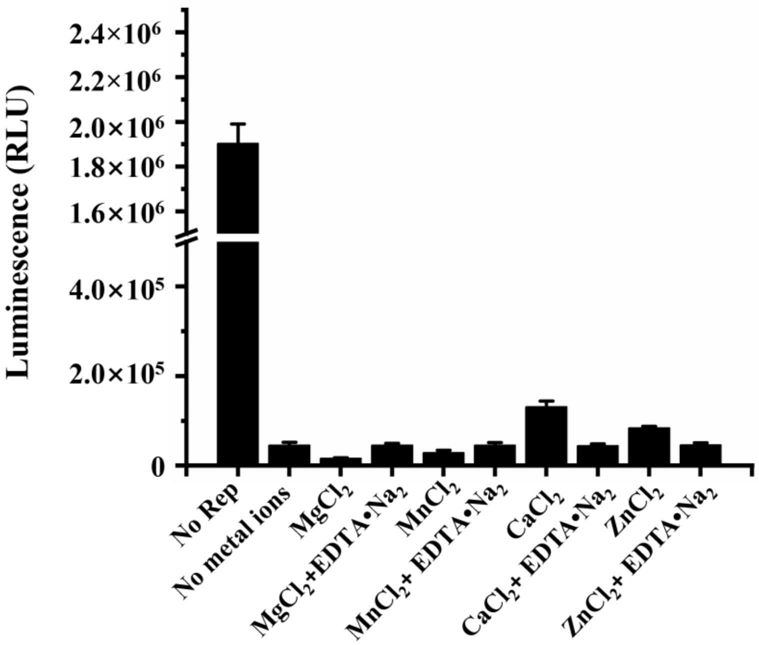

Figure S1. Verification of the effect of metal ions on the ATPase activity of Rep. In this study, 3mM $MgCl_2$, $MnCl_2$, $CaCl_2$ and $ZnCl_2$ were used in the Rep-catalyzed ATPase assays. To verify the effect of metal ions on the ATPase activity of Rep, 10 mM EDTA·Na_2 was added to the reaction buffer to completely chelate metal ions and incubated at room temperature for 10 minutes, and then the Rep was added to initiate the reaction of ATP hydrolysis. The results confirmed that $MgCl_2$ and $MnCl_2$ slightly enhanced the ATPase activity of Rep, whereas $CaCl_2$ and $ZnCl_2$ inhibited this activity.

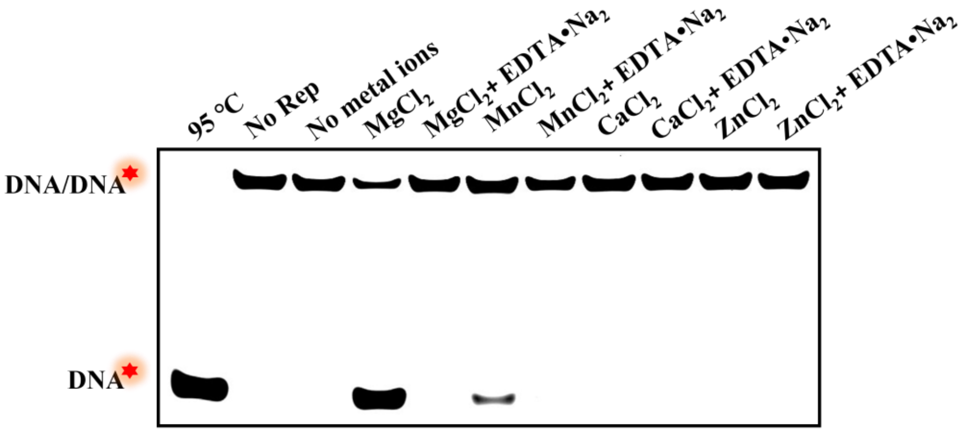

Figure S2. Divalent metal ions are crucial for the unwinding activity of Rep. 3mM $MgCl_2$, $MnCl_2$, $CaCl_2$ and $ZnCl_2$ were used in the unwinding assays, and 10 mM EDTA·Na_2 was also added to the reaction buffer to confirm the effect of metal ions on the unwinding activity of Rep.The results

showed that Rep was unable to unwind dsDNA without metal ions, and no product ssDNA was detected in the presence of $CaCl_2$ or $ZnCl_2$. Notably, the inclusion of excess EDTA to chelate metal ions completely abolished DNA unwinding activity of Rep, confirming that the observed activity was strictly dependent on the $MgCl_2$ or $MnCl_2$, and the former was more conducive to the unwinding activity of Rep. The asterisks in the figures indicate the 5′-FAM.
